# Association analysis of Epworth Sleepiness Scale (ESS) scores with serotonin transporter (5-HTT- LPR, 5-HTT-VNTR) and circadian (PER3-VNTR) genes

**DOI:** 10.5935/1984-0063.20220003

**Published:** 2022

**Authors:** Filiz Ozen, Zeynep Yegin, Zuhal Aydan Saglam, Figen Yavlal, Haydar Koc, Celal Ulasoglu

**Affiliations:** 1 Istanbul Medeniyet University, Department of Medical Genetics - Istanbul -Turkey.; 2 Sinop University, Medical Laboratory Techniques Program - Sinop - Turkey.; 3 Istanbul Training and Research Hospital, Department of Family Medicine - Istanbul- Turkey.; 4 Bahcesehir University, Department of Neurology - Istanbul - Turkey.; 5 Cankiri Karatekin University, Department of Statistics - Cankiri - Turkey.; 6 Istanbul Medeniyet University, Department of Internal Diseases - Istanbul –Turkey.

**Keywords:** Neurogenetics, Excessive daytime sleepiness (EDS), Epworth Sleepiness Scale (ESS), PER3, Serotonin transporter (5-HTT), Genetic association

## Abstract

Excessive daytime sleepiness (EDS) is a common complaint encountered in clinical practice with serious consequences both for individual and society since it can increase the ratio of motor vehicle accidents, work- related incidents, and deaths. Moreover, it also manifests less serious individual consequences. This study aimed to investigate the potential role of PER3-VNTR, 5-HTT-LPR, and 5-HTT-VNTR in terms of constituting liability to EDS. Two hundred eighteen participants (93 complaining about daytime sleepiness and 125 individuals with no serious complaint) were recruited in the study. General daytime of sleepiness was quantified with Epworth sleepiness scale (ESS). DNA extractions were performed from collected blood samples with standart salting-out procedure and genotyped. ESS scores displayed difference between individuals suffering from sleep disturbances and other individuals with values of 12.75±4.55 and 6.34±4.26, respectively. PER3- VNTR and 5-HTT-LPR genotypes did not display association with mean ESS scores. However, 5-HTT-VNTR genotypes showed significant association with mean ESS scores; individuals with 10/10 genotypes had the highest ESS score reflecting this genotype as a liability factor for EDS. We strongly recommend further studies based on circadian/serotonin pathway genes in different populations to reach to a consensus and highlight sleep genetic marker genes which then can be the future targets of pharmacological treatment studies for sleep problems.

## INTRODUCTION

Excessive daytime sleepiness (EDS) is one of the major problems in different medical departments in clinics and the nature of EDS already loaded with many clinical causes can even become more complicated with interaction of genetic variations in multiple genes.

The primate-specific variable number tandem repeat (VNTR) polymorphism (rs57875989) with in the coding region of the clock gene PERIOD3 (PER3) contains a 54-nucleotide unit which is repeated either 4 (PER3^4^ allele) or 5 times (PER3^5^ allele) in humans^1^. PER3 VNTR was found to be associated with diurnal preference; young people who were homozygous for the long repeat allele (PER3^5/5^) preferred earlier wake-up and sleep time^2^. Since then, the number of studies investigating the potential effect of PER3 VNTR in sleep studies have increased. Since 54-nucleotide repeat change is in question in the genome, we speculated that it could display a heavier effect in clinics if any, though this surely never discards the possible important effects of all other circadian genes in sleep regulation of which we could not consider in this study because of time and financial-related reasons which can be accepted as the limitation of this study.

Serotonin (5-HT) and serotonin transporter (5-HTT) genes can also have significance in sleep studies. In our study, together with circadian gene PER3, we also aimed to investigate the potential effects of two 5-HTT variations in terms of constituting liability to EDS. 5-HTT, encoded by the SLC6A4 gene plays an important role in regulating 5-HT activity via clearing released serotonin from the synaptic cleft. Inhibition of 5-HTT function by selective serotonin reuptake inhibitors was reported to be associated with poorer sleep quality and less rapid eye movement (REM) sleep^3,4^.

Briefly, we aimed to investigate the potential role of PER3- VNTR, 5-HTT-LPR, and 5-HTT-VNTR in terms of constituting liability to EDS and thus represent a molecular genetics perspective with these genes in circadian rhythm and serotonin network.

## MATERIAL AND METHODS

### Subjects

We recruited a total of 218 unrelated Turkish individuals (93 complaining about daytime sleepiness and 125 individuals with no serious complaint). Participants with disorders that can affect sleepiness evaluation such as having hypo/hypertension, diabetes mellitus, thyroid function disorders, depression were excluded. The protocol of this study was approved by the Local Committee of Medical Ethics at Istanbul Medeniyet University (Approval number: 2019/0299).

### Measures

#### Sleepiness assessment

All of the participants (both the patients and controls) completed ESS5 *questionnaire* which is a valid measurement of sleep propensity in adults, between office hours 08.00 – 17.00. ESS is an 8-item method each scored with a degree of severity ranging from 0 to 3, and thus giving a total score of between 0 and 24 for each subject to distinguish individuals in terms of daytime sleepiness. ESS scores >10 may reflect sleepiness propensity and consultation to sleep medicine specialist to diagnose and treat the possible causes of sleepiness. However, in order to calculate the cut-off value of the ESS score, the 75th percentile of the current sample was taken into account to improve the sensivity of our results.

### Genotyping

DNA extraction from blood samples was made according to the salting-out procedure^6^. PER3 VNTR, 5-HTT-LPR and 5-HTT-VNTR variants were determined with PCR technique. Primer sequences were previously described: PER3-F: 5′-TGT CTT TTC ATG TGC CCT TAC TT-3′, PER3-R: 5-TGT CTG GCA TTG GAG TTT GA-3′; 5-HTT-LPR-F: 5′-GGC GTT GCC GCT CTG AAT TGC-3′, 5-HTT-LPR-R: 5’-GAG GGA CTG AGC TGG ACA ACC AC-3′; 5-HTT-VNTR-F: 5′-GTC AGT ATC ACA GGC TGC GAG-3′, 5-HTT-VNTR-R: 5′- TGT TCC TAG TCT TAC GCC AGT G-3′^7,8^.

PER3 PCR reaction was carried out in a 25 µl total volume containing ~150 ng genomic DNA, 0.5 µM of each primer, 0.2 mM of each dNTP, 2 mM MgCl_2_, 1x Taq polymerase buffer, and 1.5 U Taq polymerase (Thermo Fisher Scientific, MA, USA). PCR conditions were as follows: 35 cycles of denaturation for 40 sec at 94°C, annealing for 45 sec at 55°C and extension for 45 sec at 70°C. A pre-denaturation step for 6 min at 94°C was included before the cycles and a final extension step for 12 min at 70°C completed the reaction. Amplicons were analyzed on agarose gel electrophoresis to distinguish the 5-repeats allele (401 bp) from the 4-repeats allele (347 bp), generated by the ins/del polymorphism.

PCR reaction conditions for 5-HTT-LPR in a 25 µl volume were as follows: ~150 ng genomic DNA, 1 µM of each primer, 0.2 mM of each dNTP, 1 mM MgCl_2_, 1x Taq (NH_4_)_2_SO_4_ buffer, and 2 U Taq polymerase (Thermo Fisher Scientific, MA, USA). PCR cycling conditions: 40 cycles of denaturation for 30 sec at 95°C, annealing for 30 sec at 62°C and extension for 1 min at 72°C. A pre-denaturation step for 2 min at 95°C and a final extension step for 7 min at 72°C were included. Agarose gel electrophoresis of amplicons allowed to detect the fragments of 484 bp (S allele) and 528 bp (L allele). PCR reaction conditions for 5-HTT-VNTR were same of the 5-HTT-LPR with the difference of MgCl_2_ concentration which was increased to 2.5 mM in latter case. PCR cycling conditions of 5-HTT VNTR included a pre-denaturation step for 5 min at 95°C, 35 cycles of denaturation for 45 sec at 95°C, annealing for 45 sec at 56°C, extension for 1 min at 72°C, and a final elongation step of 10 min at 72°C. PCR products were loaded on 3% ultra pure agarose gel, stained with ethidium bromide, electrophoresed and analyzed with gel documentation system (SYNGENE Ingenius 3, England). 10-repeats allele (10/10) produced the fragment size of 267 bp while 12-repeats allele (12/12) produced 300 bp. Heterozygotes (10/12) resulted with both bands.

### Statistical analysis

Genotype frequencies were assessed for Hardy– Weinberg equilibrium (HWE) by χ2 test. Statistical analysis was done using statistical package SPSS 22 software. The relationship of ESS scores between daytime sleepiness cases and controls was evaluated with Student’s t-test. The possible associations between genotypes **were** analyzed and ESS scores were calculated by computing the odds ratio (OR) and 95% confidence intervals (95% CI) and one-way analysis of variance (ANOVA). Also, bonferroni correction was made for pairwise comparisons when there was a significant relationship as a result of ANOVA. The statistical level of significance was defined as p<0.05 (When relevant statistical analyses were performed, genotypes were adjusted for age variable).

## RESULTS

The mean ages of daytime sleepiness cases and controls were 48.22±15.94 and 42.17±14.89, respectively. Since obesity is an important risk factor for daytime sleepiness, BMI values were also assessed. BMI value of controls was 28.45±21.99 while the same value of cases increased to 32.87±46.40 which is over the threshold 30 kg/m^2^ that is accepted as an obesity indicator. ESS scores between cases and controls were significantly different as 12.75±4.55 and 6.34±4.26, respectively (Table 1).

**Table 1:** ESS scores, ages, and BMI values of participants.

Group	n	ESS score	Age	BMI
**Cases**	93	12.75±4.55	48.22±15.94	32.87±46.40
**Controls**	125	6.34±4.26	42.17±14.89	28.45±21.99
**t**		10.686	2.891	0.940
**p value**		0.000	0.004	0.348

Genotype frequencies were assessed for Hardy–Weinberg equilibrium (HWE) by χ2 test and there was no deviation from HWE for the genes investigated. The effects of PER3 VNTR, 5-HTT-LPR, and 5-HTT-VNTR genotypes in terms of constituting liability to EDS were first determined with odds ratio (OR) and 95% confidence intervals (95% CI) by using ESS score ≥12 as a cut-off point. Then, a comparison excluding grouping and based on mean ESS scores **was** also conducted with ANOVA.

PER3 VNTR genotypes did not show difference between high sleepiness (ESS≥12) and normal sleepiness (ESS<12) groups. PER3 genotypes did not show association with mean ESS scores without grouping, either (Table 2 and Table 3).

**Table 2:** Odds ratios (OR) and 95% confidence intervals (CI) for PER3 VNTR genotyping.

Genotype	N	High Sleepiness	Normal Sleepiness	OR (95% CI)	p-value for OR
**4R**	87	25	62	0.863 (0.341-2.184)	0.755
**4/5R**	100	23	77	1.211 (0.460- 2.950)	0.748
**5R**	31	8	23	Reference	
**p-value for** *χ*^2^ Allele			0.67		
**4R**	274	73	201	0.873 (0.557-1.368)	0.552
**5R**	162	39	123	Reference	
**p-value for** *χ*^2^			0.553		

**Table 3:** Comparison of PER3 VNTR genotypes and mean ESS scores.

Genotype	N	Mean ESS scores (SD)	F	p value
**4R**	87	9.103 (5.618)	0.361	0.698
**4/5R**	100	8.830 (5.401)		
**5R**	31	9.774 (4.917)		

5-HTT-LPR genotypes did not differ between high sleepiness (ESS≥12) and normal sleepiness (ESS<12) groups. There was no association with 5-HTT-LPR genotypes and mean ESS scores without grouping (Table 4 and Table 5).

**Table 4:** Odds ratios (OR) and 95% confidence intervals (CI) for 5-HTTLPR genotyping.

Genotype	N	High Sleepiness	Normal Sleepiness	OR (95% CI)	p-value for OR
**L/L**	50	9	41	2.000 (0.805-4.967)	0.135
**L/S**	109	29	80	1.211 (0.602-2.435)	0.591
**S/S**	59	18	41	Reference	
**p- value for** *χ*^2^ Allele			0.314		
**L**	209	47	162	1.383 (0.8961-2.134)	0.143
**S**	227	65	162	Reference	
**p- value for** *χ*^2^			0.142		

**Table 5:** Comparison of 5-HTT-LPR genotypes and mean ESS scores.

Genotype	N	Mean ESS scores (SD)	F	p value
**L/L**	50	8.2 (4.77)	0.98	0.377
**L/S**	109	9.17 (5.48)		
**S/S**	59	9.62 (5.77)		

The association between 5-HTT-VNTR and ESS scores **was** also evaluated both with odds ratios (OR) and 95% confidence intervals (CI) and ANOVA analysis. ANOVA results showed that there was a significant association between 5-HTTVNTR genotypes and ESS scores. Individuals with 10/10 genotype displayed the highest mean ESS score reflecting that this genotype **displayed** liability for daytime sleepiness (Table 6 and Table 7).

**Table 6:** Odds ratios (OR) and 95% confidence intervals (CI) for 5-HTT-VNTR genotyping.

Genotype	N	High Sleepiness	Normal Sleepiness	OR (95% CI)	p-value for OR
**10/10**	31	13	18	0.467 (0.203-1.073)	0.073
**10/12**	76	15	61	1.372 (0.675-2.787)	0.382
**12/12**	111	28	83	Reference	
**p-value for** χ^2^			0.058		
**Allele**					
**10**	138	41	97	0.740 (0.471-1.163)	0.192
**12**	298	71	227	Reference	
**p-value for** χ^2^			0.191		

**Table 7:** Comparison of 5-HTT-VNTR genotypes and mean ESS scores.

Genotype	N	Mean ESS scores (SD)	F	p value
**10/10**	31	11.645 (5.148)	4.601	0.011
**10/12**	76	8.236 (4.904)		
**12/12**	111	8.927 (5.632)		

The recapitulated overview explaining the possible relations between investigated genes and mean ESS scales was shown in Table 8.

**Table 8:** Recapitulated version between mean ESS scales and investigated genes.

	Genotype	N	Mean ESS scores (SD)	F	p value
PER3 VNTR genotypes	4R	87	9.103 (5.618)	0.361	0.698
	4/5R	100	8.830 (5.401)		
	5R	31	9.774 (4.917)		
5-HTT-LPR genotypes	L/L	50	8.2 (4.77)	0.98	0.377
	L/S	109	9.17 (5.48)		
	S/S	59	9.62 (5.77)		
5-HTT-VNTR genotypes	10/10	31	11.645 (5.148)	4.601	0.011
	10/12	76	8.236 (4.904)		
	12/12	111	8.927 (5.632)		

## DISCUSSION

One of the shared symptoms of patients to consult clinics is excessive daytime sleepiness (EDS) and the diagnosis and the treatment of sleep disorders may necessitate a multi-disciplinary team approach with the inclusion of different departments such as family medicine, neurology, psychiatry, gastroenterology, pulmonology, general surgery etc. Molecular genetic approaches may also have a substantial impact to gain an insight about genetic drivers. EDS presents many serious consequences such as annually contributing to more than 100,000 motor vehicle incidents, 71,000 personal injuries and 1,500 deaths^9^. Besides this very important scheme, EDS also leads to somehow less serious individual consequences such as personal productivity at school/ work, concentration, memory and mood problems^10^. There are a various number of reasons for EDS such as insufficient sleep (the first condition for identifying), sleep apnoea and sleep-disordered breathing, periodic limb movement disorder (PLMD) and restless legs syndrome (RLS), some neurological disorders such as Parkinson’s disease, multiple sclerosis, stroke, epilepsy, CNS tumors, metabolic problems such as obesity, anemia and hypothyroidism, some psychiatric disorders such as depression, anxiety, and post-traumatic stress disorder, other organic diseases such as congestive heart failure, chronic renal failure, liver failure, medications such as antidepressants, anxiolytics, pain medications, and antiepileptics, drug and alcohol use^11,12^.

One of the simple tools to assess EDS in clinical practice is the Epworth Sleepiness Scale (ESS) developed by Johns (1991)^5^. ESS is an appropriate 8-item method to subjectively evaluate how likely individuals are prone to fall sleep in a variety of common situations. In the study of Zwahlen et al. (2016)^13^ which was conducted in private and professional drivers having a medical appointment in the Department of Traffic Sciences at the Institute of Forensic Medicine, University of Bern, nearly one out of six of the questioned drivers admitted to fall sleep while driving a motor vehicle and these participants’ ESS scores were found significantly higher. In the study of Quaranta et al. (2016),^14^ sleepiness and predictivity of obstructive sleep apnea in drivers were evaluated both with the Driver Sleepiness Score (DSS) and ESS and the combination of both questionnaires was offered for the detection of all OSA severity levels with high accuracy. In an other study, ESS and apnea presence were offered as the best predictors of road accidents^15^. The association between higher scores of ESS and sleepy driving was also reported in the study of Gonçalves et al. (2015)^16^. Çetinoğlu et al. (2015)^17^ reported the higher scores of ESS for drivers with a history of road traffic accident (RTA). In a very recent nurse-based surveillance study conducted in Turkey, it was reported that most of the nurses experienced occupational accidents and those who had these accidents had higher mean ESS scores^18^.

In the study of Viola et al. (2012)^19^, homozygosity for the longer allele (PER3^5/5^) was found to be associated with phase-advance in the circadian melatonin profile and earlier melatonin peak occurrence within sleep episode. PER3 VNTR’s effect was analyzed in night-shift workers in terms of its association with sleepiness and maladaptive circadian phase and the researches found that while people with PER3^4/4^ genotypes showed sleepiness within normal limits, PER3^-/5^ workers displayed pathological levels of sleepiness which is important in terms of occupational and automotive accidents^20^. The similar finding was also reported in the study of Cheng et al. (2018)^21^; PER3^4/4^ individuals were more susceptible to insomnia associated with trait sleep reactivity, while individuals with PER3^5/−^ genotype were prone to circadian misalignment associated insomnia. On the other hand, some studies report the lack of association between PER3 VNTR and diurnal preference/sleep quality^22,23,24,25^. We can not rule out the possibility that PER3 VNTR may play a significant role in circadian/sleep phenotypes in some populations but deciding about whether PER3 VNTR is a good candidate as a sleep genetic marker or not merits further investigations in different populations. In our study, in concordance with some of the above studies, we did not find an association between PER3 VNTR and daytime sleepiness.

Two variations in 5-HTT gene (5-HTT-LPR and 5-HTT-VNTR) have the potential to affect sleep regulation. S allele of 5-HTT-LPR was reported to moderate sleep disturbance as a response to chronic stress^26^. Yue et al. (2008)^27^ analyzed 5-HTT-LPR and 5-HTT-VNTR genotypes in patients with sleep apnea syndrome (SAS) and though S/L alleles of 5-HTT-LPR did not differ between SAS patients and healthy controls, they offered 10 allele of 5-HTT-VNTR as a susceptibility factor for SAS pathogenesis. These results are in coherent with our results; though we could not find an association between EDS and 5-HTT-LPR genotypes, 10/10 genotype of 5-HTT-VNTR in our study population was a risk factor for EDS. In contrast with the study of Yue et al. (2008)^27^, an other study reported S allele of 5-HTT-LPR as a risk factor for imsomnia patients^28^. Our results are also consistent with the study of Chen et al. (2013)^8^ who did not find difference between OSAS patients and control subjects in terms of 5-HTT-LPR but offered 10/10, 10/12 genotypes and 10 allele frequency of 5-HTT-VNTR as risk factors. The authors also offered significant differences in L allele of 5-HTT-LPR in only male patients and male controls. L allele of 5-HTT-LPR was proposed as an important risk factor for the greater severity of OSA in older adults^29^. van Dalfsen et al. (2019)^30^ did not find an association between 5-HTT-LPR genotype status and insomnia. Based upon the literature studies related with sleep conducted with 5-HTT-LPR and 5-HTT-VNTR genotypes up to now, it is somehow clear that though the effect of 5-HTT-LPR is prone to be heterogenous, 5-HTT-VNTR’s effect seems to be steadier. It was reported that the most common frequent alleles of 5-HTT-VNTR containing 10 and 12 repeats act as transcriptional regulators with allele-dependent differential enhancer-like properties, which may result with the differences in serotoninergic activity^31^. Surely, we could evaluate a limited number of SNPs which can be recommended to be extended in future sleepiness studies. Moreover, it is notably important to emphasize that genome-wide association studies (GWAS) are of significant power to identify new variants. Recently, Wang et al., (2019)^32^ analyzed 452,071 participants of European genetic ancestry in the UK Biobank and identified 42 loci for self-reported daytime sleepiness in these individuals, with enrichment for genes expressed in brain tissues and in neuronal transmission pathways. As being the largest GWAS of self-reported daytime sleepiness, this study is very powerful. Nevertheless, the authors explained several limitations; lack of frequently used measures of daytime sleepiness such as the Epworth Sleepiness Scale (ESS) or Maintenance of Wakefulness Test, and the homogeneity of their cohort (Only individuals of European ancestries aged 40–69 years old in the UK). Therefore, the analyses of the candidate sleep gene markers in different populations, preferably with larger individual numbers (which is also a major limitation of our study) are also required.

A better understanding of EDS reasons and elucidating possible items of both clinical and genetic is significant to improve public health and prevent accidental situations. As a recapitulation of the essential points in our study, we can say that though PER3 VNTR and 5-HTT-LPR genotypes were not susceptibility factors for EDS, 5-HTT-VNTR 10/10 genotype seems to serve as a risk factor for EDS in our study population. Future replication studies and more variation analyses in circadian/serotonin pathway genes seem a prerequisite to draw more precise conclusions.
